# The Effect of Disruption of Prefrontal Cortical Function with Transcranial Magnetic Stimulation on Visual Working Memory

**DOI:** 10.3389/fnsys.2015.00169

**Published:** 2015-12-16

**Authors:** Elizabeth S. Lorenc, Taraz G. Lee, Anthony J.-W. Chen, Mark D’Esposito

**Affiliations:** ^1^Helen Wills Neuroscience Institute, University of CaliforniaBerkeley, Berkeley, CA, USA; ^2^Department of Psychology, University of MichiganAnn Arbor, MI, USA; ^3^Department of Neurology, VA Northern California Healthcare SystemMartinez, CA, USA; ^4^Department of Neurology, University of CaliforniaSan Francisco, San Francisco, CA, USA; ^5^Department of Psychology, University of CaliforniaBerkeley, Berkeley, CA, USA

**Keywords:** visual working memory, functional magnetic resonance imaging, transcranial magnetic stimulation, executive function, selective attention, prefrontal cortex

## Abstract

It is proposed that feedback signals from the prefrontal cortex (PFC) to extrastriate cortex are essential for goal-directed processing, maintenance, and selection of information in visual working memory (VWM). In a previous study, we found that disruption of PFC function with transcranial magnetic stimulation (TMS) in healthy individuals impaired behavioral performance on a face/scene matching task and decreased category-specific tuning in extrastriate cortex as measured with functional magnetic resonance imaging (fMRI). In this study, we investigated the effect of disruption of left inferior frontal gyrus (IFG) function on the fidelity of neural representations of two distinct information codes: (1) the stimulus category and (2) the goal-relevance of viewed stimuli. During fMRI scanning, subjects were presented face and scene images in pseudo-random order and instructed to remember either faces or scenes. Within both anatomical and functional regions of interest (ROIs), a multi-voxel pattern classifier was used to quantitatively assess the fidelity of activity patterns representing *stimulus category*: whether a face or a scene was presented on each trial, and *goal relevance*, whether the presented image was task relevant (i.e., a face is relevant in a “Remember Faces” block, but irrelevant in a “Remember Scenes” block). We found a reduction in the fidelity of the *stimulus category* code in visual cortex after left IFG disruption, providing causal evidence that lateral PFC modulates object category codes in visual cortex during VWM. In addition, we found that IFG disruption caused a reduction in the fidelity of the *goal relevance* code in a distributed set of brain regions. These results suggest that the IFG is involved in determining the task-relevance of visual input and communicating that information to a network of regions involved in further processing during VWM. Finally, we found that participants who exhibited greater fidelity of the *goal relevance* code in the non-disrupted right IFG after TMS performed the task with the highest accuracy.

## Introduction

Since the human brain has an inherently limited capacity for information processing and working memory (Cowan et al., 2005), it is crucial that relevant information in the environment be filtered from the myriad of visual details that are unimportant, and often detrimental, to the task at hand (Vogel et al., 2005). It is proposed that biased competition among representations of features in the visual field is resolved via both top-down and bottom-up signals, with the top-down influence likely guided by an “attentional template” maintained in working memory (Desimone and Duncan, 1995; Desimone, 1998). There is increasing evidence that the prefrontal cortex (PFC) is one source of these top-down signals which are essential for the privileged processing and maintenance of goal-relevant visual information within extrastriate cortex (Miller and D’Esposito, 2005; Bressler et al., 2008; Sreenivasan et al., 2014b; D’Esposito and Postle, 2015). Consistent with this view, we recently demonstrated that selective attention alters the tuning of stimulus category representations in extrastriate cortex, while the lateral PFC codes for the current task goal (i.e., “Remember Faces, Ignore Scenes”; Chen et al., 2012).

Successful filtering of relevant visual information is essential for the prioritized storage of that information in working memory for later use, and information in working memory can further guide selective attention. Evidence for top-down modulatory processes shaping neural activity has been found throughout different stages of working memory (Gazzaley and Nobre, 2012): stimulus anticipation (e.g., Bressler et al., 2008; Puri et al., 2009; Esterman and Yantis, 2010), sensory processing and gating of information to be encoded into working memory (e.g., Gazzaley, 2011; Kok et al., 2012), prioritization and manipulation of memory representations (e.g., Nee and Jonides, 2009; Tamber-Rosenau et al., 2011; Kuo et al., 2014), and memory retrieval (Nobre et al., 2008).

Lesion studies provide evidence for the role of frontal cortex as one source of top-down signals that can modulate processing in sensory regions during working memory. Fuster et al. (1985) were the first to investigate the effect of PFC cooling on spiking activity in inferotemporal (ITC) neurons during a delayed-match-to-sample task. During the delay period—when persistent stimulus-specific ITC activity is observed—cooling caused attenuated spiking profiles and a loss of stimulus-specificity in ITC neurons. In humans, Barceló et al. (2000) found that lateral PFC lesions caused reduced extrastriate activity in the lesioned hemisphere and correspondingly lateralized behavioral deficits. In addition, Sauseng et al. (2011) found that TMS disruption of right frontal eye field function in healthy participants impaired the shifting of visuospatial attention, and yielded corresponding changes in electrocorticographic measures of neural dynamics. Finally, we previously demonstrated that TMS disruption of lateral PFC function impaired performance on a face/scene matching task, while reducing category-specific tuning in extrastriate cortex (Lee and D’Esposito, 2012). These results provide important causal evidence for the role of the PFC in shaping the tuning of information processed in extrastriate cortex, and provide insight into the dynamic nature of top-down modulation of visual areas by the PFC in accordance with task goals.

The present study uses a set of multi-voxel pattern classification analyses to further investigate the effects of PFC disruption on the neural representation of stimulus category and goal-relevance information codes. Immediately after continuous theta-burst TMS to the left inferior frontal gyrus (IFG) or a control region (left somatosensory cortex), participants underwent MRI scanning while performing a face/scene matching task, in which the relevant stimulus category (faces or scenes) varied by block. With this approach, we investigated the effect of frontal cortex disruption on the fidelity, as indexed by decoding accuracy, of two distinct types of visual working memory (VWM) representations: (1) stimulus category: whether a face or a scene was presented on each trial and (2) goal relevance, whether the presented image was task relevant (i.e., a face is relevant in a “Remember Faces” block, but irrelevant in a “Remember Scenes” block). First, we hypothesized that disruption of top-down control signals emanating from the left IFG would reduce the fidelity of the *stimulus category* code within extrastriate cortex. Second, given that PFC likely maintains a code for goal relevance, we hypothesized that PFC disruption would reduce the fidelity of this information code in this disrupted PFC region, as well as other areas that *depend* on information from this disrupted region.

## Materials and Methods

Analyses were applied to unpublished and published data (Lee and D’Esposito, 2012).

### Participants

Data from 24 participants (8 male, age range 18–38) were analyzed in this study. Data from 15 participants have not been previously published and data from nine participants were published in Lee and D’Esposito (2012). Although the Lee and D’Esposito study originally included 12 participants, three of those participants were excluded due to methodological issues specific to the current analyses. All procedures were approved by the UC Berkeley Committee for the Protection of Human Subjects, and participants gave their written informed consent before the study and were compensated monetarily for their participation.

### Cognitive Task

**Figure 1:**
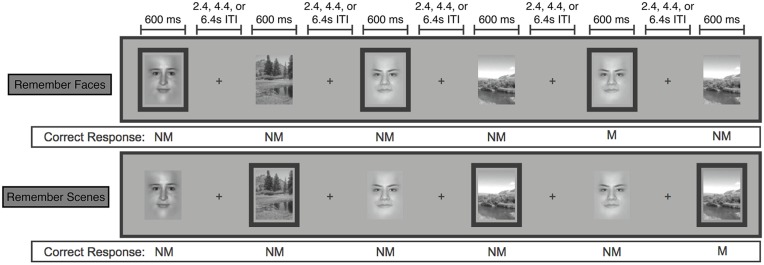
**Schematic of the face/scene matching task.** Stimuli from the task-relevant category were separated by 0–3 intervening non-relevant images. Task-relevant images are outlined here, but outlines were not shown to participants. (NM = nonmatch, M = match). Figure modified from Lee and D’Esposito (2012).

In the MRI scanner, participants viewed a series of pseudo-randomly interleaved face and natural scene images in a jittered, event-related design with 3, 5 or 7 s in between the onset of each 600 ms stimulus presentation (Chen et al., 2008, 2011; Figure 1). In separate scanning runs, participants performed a 1-back matching task within the faces only (“Remember Faces”) or scenes only (“Remember Scenes”) behavioral conditions. Participants responded to each image with a button press indicating a 1-back “match” or “non-match” within the relevant category, and they also indicated “non-match” for all images of the irrelevant category. Participants also completed runs in which they were required to perform the 1-back matching task within both stimulus categories simultaneously, and runs in which they simply categorized each stimulus as a face or a scene, but these conditions were not of interest for the present analyses. Each participant completed five 20-trial 2 min runs of each behavioral condition, each of which contained four matches. To ensure that the pattern classification analyses were balanced and unbiased, both “match” and “non-match” and correct and incorrect trials were included in each of the following analyses.

### Transcranial Magnetic Stimulation

Detailed descriptions of the TMS methods used in this study have been published previously (Lee and D’Esposito, 2012). Immediately before each of two MRI scan sessions, nine participants underwent 40 s of continuous theta burst TMS, either to the left inferior frontal gyrus (“IFG TMS”) or to the left postcentral gyrus (“Control TMS”).

There was an average of 8 days between the IFG TMS and Control TMS scan sessions, with a range of 2–18 days. After the exclusion of three participants of the original 12 (see “Participants” Section), a total of two participants first underwent IFG TMS followed by Control TMS, and seven first underwent Control TMS. Given that each participant completed five 20-trial runs of each behavioral condition in an initial functional magnetic resonance imaging (fMRI) scan session prior to the two TMS/fMRI scans, it is unlikely that order effects account for the findings reported below. Moreover, re-analysis of the data accounting for order found no evidence of a systematic difference in TMS effects in the two order groups.

Left IFG TMS targets were defined functionally in a separate scan session with the same behavioral task, using a statistical contrast of all attended images vs. all ignored images, regardless of stimulus type, across all task conditions. Left postcentral gyrus TMS targets were anatomically defined using the Duvernoy brain atlas (Duvernoy, 1999) as a reference, and drawn as spheres with a radius of 5 mm centered 10 mm away from the midline and 5 mm from the top edge of the brain. TMS sites were identified in native space for each participant, and the corresponding MNI coordinates are listed in Table 1.

**Table 1:** **MNI coordinates of left IFG and control (left postcentral gyrus) TMS sites for each individual subject**.

	Left IFG MNI Coord. (mm)			Left postcentral gyrus MNI coord. (mm)		
Subject number	*x*	*y*	*z*	*x*	*y*	*z*
1	−53	−3	20	−10	−36	73
2	−51	9	13	−14	−33	70
3	−50	1	14	−10	−34	73
4	−45	8	8	−15	−37	69
5	−62	−10	19	−10	−35	67
6	−48	9	21	−9	−37	69
7	−42	2	31	−15	−41	65
8	−50	6	25	−9	−36	66
9	−46	10	20	−11	−41	67

Continuous theta burst TMS, which provides localized activity disruption for up to 60 min after stimulation (Huang et al., 2005), consists of 50 Hz TMS pulse triplets administered every 200 ms (5 Hz) for a total duration of 40 s.

### Functional MRI Acquisition and Preprocessing

MRI data were acquired in the UC Berkeley Henry H. Wheeler, Jr. Brain Imaging Center with a Siemens TIM/Trio 3T MRI scanner with a 12-channel receive-only head coil. Functional data were obtained using a one-shot T2*-weighted echoplanar imaging (EPI) sequence sensitive to blood oxygenation level-dependent (BOLD) contrast (TR, 1000 ms; TE, 32 ms; field of view, 230 mm; matrix size, 64 × 64; in-plane resolution, 3.5 × 3.5 mm). Each functional volume contained 18 contiguous 5 mm-thick axial slices separated by a 0.5 mm interslice gap. Whole-brain MP Flash T1-weighted scans were acquired for anatomical localization and normalization.

Functional MRI data were then subject to standard preprocessing with AFNI (Cox, 1996) and custom Matlab (v2011b, The MathWorks, Inc., Natick, MA, USA) scripts. Motion correction and volume registration of each EPI run to the anatomical scan was carried out in a single resampling step by align_epi_anat.py (Saad et al., 2009), by first aligning the mean of the middle EPI to the anatomical data and then aligning each volume to that mean EPI with a 12-parameter affine registration. Next, AFNI’s 3dDeconvolve tool was used to compute an ordinary least squares regression with 15 double gamma canonical hemodynamic response function regressors: eight stimulus regressors, one for each stimulus-category—memory-condition combination (i.e., a face in “Remember Faces”, a scene in “Remember Faces”, etc.), six motion parameter regressors (*x*, *y*, *z*, roll, pitch, yaw), and a quintic polynomial baseline regressor. Then, the resulting β-weighted estimated baseline component (motion + polynomial baseline) was calculated with AFNI’s 3dSynthesize tool and subtracted from the original time series. Finally, each run was *z*-scored temporally, voxel-wise, in preparation for multi-voxel pattern analysis (MVPA).

### Multi-Voxel Pattern Classification Analyses

In all of the following pattern classification analyses, we determined the fidelity of neural codes representing the category of each stimulus (face or scene) which we call the “stimulus category” code and the relevance of each stimulus to the current task goal (“remember faces” or “remember scenes”) which we call the “goal relevance” code.

#### Stimulus Category Code

A classifier was trained to distinguish multi-voxel activity patterns evoked by the presentation of a face from those evoked by presentation of a scene, regardless of the relevance of the stimulus category to the current task condition (Chen et al., 2011). Based on our unpublished data which found that the coding of stimulus category information peaks just over 5 s after stimulus onset, this code was examined using BOLD signal from the EPI volume collected 5–6 s post stimulus onset.

#### Goal Relevance Code

A classifier was trained to distinguish multi-voxel activity patterns representing the relevance of each stimulus to the current task set (i.e., Relevant: a face in “Remember Faces” or a scene in “Remember Scenes”, vs. Irrelevant: a scene in “Remember Faces” or a face in “Remember Scenes”). Based on our unpublished data which found that the coding of goal relevance information peaks later than the stimulus category code, about 6.5 s after stimulus onset, this code was examined using the BOLD signal from the EPI volume collected 6–7 s post stimulus onset.

#### Regions of Interest—Anatomical

*A priori* regions of interest (ROIs) were defined anatomically, by first registering each participant to MNI152 space (Grabner et al., 2006) and then back-projecting masks from the AAL atlas (Tzourio-Mazoyer et al., 2002) into the participant’s native space. Anatomical ROIs included: left and right middle frontal gyrus (MFG), IFG (which includes pars opercularis, pars triangularis, and pars orbitalis), and extrastriate cortex (parahippocampal, lingual, and fusiform gyri).

### Regions of Interest—Functional

Functional ROIs were created from a dataset of 24 participants. This included previously unpublished data from 15 participants, and published data from nine participants who performed the behavioral task in the scanner prior to undergoing TMS (Lee and D’Esposito, 2012). To create “stimulus category” and “goal relevance” ROIs, we conducted whole-brain Gaussian Naïve Bayes searchlight analyses separately within each participant using the Searchmight toolbox (Pereira and Botvinick, 2011). Each 27-voxel cubic searchlight was iteratively moved throughout every voxel in the brain, following a leave-one-run-pair-out (one “Remember Faces” and one “Remember Scenes” run) cross-validation structure. The mean classification accuracy across all five cross-validation folds was assigned to the center voxel of each searchlight position, forming a stimulus category and a goal-relevance accuracy map for each participant. These accuracy maps were then spatially smoothed with an 8 mm FWHM Gaussian kernel, warped to MNI space, and then entered into a second-level group analysis in which the mean decoding accuracy at each voxel was tested against 50% chance accuracy with a one-sample *t*-test. The resulting t-map was used to threshold the mean across-subjects accuracy map at a stringent false-discovery-rate-corrected alpha level of 0.0001.

### ROI Pattern Classification Analysis

Within anatomical (MFG, IFG, and extrastriate cortex) and functional ROIs, a regularized logistic regression classifier (Princeton Multi-Voxel Pattern Analysis toolbox v1.1; http://code.google.com/p/princeton-mvpa-toolbox/) was used to test for TMS-induced changes in the fidelity of codes representing stimulus category and goal relevance. All MVPA analyses were run with an iterative cross-validation procedure in which all but one pair of runs (one “Remember Faces” + one “Remember Scenes”) were used to train the classifier, and the held-out pair were then used as a test set to assess classifier accuracy. Non-parametric permutation tests were used to test for above-chance classification, as well as to test for significant differences between information code fidelity (indexed by classifier accuracy) in the two TMS conditions. More specifically, 1000 sets of permuted class labels were pre-generated, following the cross-validation structure of the original analysis. Then, single-subject null classifier accuracy distributions were created separately for each ROI and TMS condition, each time using the same 1000 sets of permuted class labels. Finally, the single-subject classifier accuracies for each of the 1000 sets of permuted labels were averaged across subjects, to create a null distribution of mean classifier accuracies against which to test the observed mean classifier accuracies.

To test whether classification accuracy was significantly above chance within each ROI and TMS condition, we calculated the fraction of the null classifier accuracy distribution that exceeded the observed classifier accuracy. This allowed for the calculation of empirical *p*-values for each ROI and TMS condition, which were then Bonferroni corrected for multiple comparisons. Finally, we tested whether classification accuracy decrements after IFG TMS as compared to control TMS were greater than what would be expected by chance. First, a null “TMS condition difference” distribution was created for each ROI by subtracting the classifier accuracy in each permutation of the IFG TMS condition data from the classifier accuracy in the matching Control TMS condition permutation, and averaging across all eight participants. The *p*-value of the resulting TMS condition difference within each ROI was calculated as the fraction of the null TMS condition difference distribution that exceeded the true TMS condition difference. Finally, these empirical *p*-values were Bonferroni corrected for multiple comparisons. These analyses were repeated for each information code.

### Whole-Brain Searchlight Classification Analysis

This analysis was designed to investigate whether brain regions outside our initially hypothesized regions also code stimulus category and/or goal relevance, and to test whether the fidelity of these information codes are affected by left IFG disruption with TMS. Using the Searchmight toolbox (Pereira and Botvinick, 2011), we conducted a whole-brain Gaussian Naive Bayes searchlight analysis separately within each participant and TMS condition (IFG TMS, control TMS). Each 125-voxel cubic searchlight was iteratively moved throughout every voxel in the brain, with the mean classification accuracy across all cross-validation folds assigned to the center voxel of the searchlight. This yielded one accuracy map per TMS condition per participant, and each participant’s IFG TMS accuracy map was then subtracted from the control TMS accuracy map to create a “true TMS condition difference” accuracy map. The resulting difference maps were normalized to MNI space, spatially smoothed with an 8 mm FWHM Gaussian kernel, and then entered into a non-parametric group analysis similar to that proposed by Stelzer et al. (2013). More specifically, 100 sets of permuted labels were generated, and used to create 100 null searchlight accuracy maps per participant. Then, 100,000 average group maps were created via a bootstrapping procedure: on each of the 100,000 iterations, 1 of the 100 maps was drawn randomly with replacement from each participant, and the resulting maps were averaged across participants. Next, the “true TMS condition difference” mean accuracy map was thresholded voxelwise, with each voxel only passing the threshold if its true value exceeded 99.5% of the 100,000 values in the null distribution. Finally, we performed a cluster correction procedure in which the cluster size threshold was determined empirically from our 100,000 null group maps. First, the size of the largest contiguous cluster (comprised of voxels sharing faces, not just edges or corners) in each of the 100,000 null group maps was calculated and recorded. Finally, any clusters in the “true TMS condition difference” map larger than 99.5% of the maximum clusters from the null maps were considered significant.

## Results

These analyses were applied to data previously published (Lee and D’Esposito, 2012). In the previous article, univariate, spatial similarity, and functional connectivity analyses indicated that left IFG disruption reduced category-specific tuning in extrastriate cortex and impaired performance on a face/scene matching task. In addition, activity in the non-disrupted right IFG, and connectivity between this region and extrastriate cortex, predicted resistance to behavioral impairment from left IFG disruption.

In the current study, to assess the effects of lateral PFC disruption on the neural representation of the active maintenance of information codes during working memory, we examined multi-voxel patterns of activity within* a priori* anatomical and functional ROIs as well as across the whole brain. Specifically, we examined two distinct types of representations: (1) *stimulus category*—whether a face or a scene was presented on each trial of a face/scene matching task and (2) *goal relevance—*whether the presented image was task relevant (i.e., a face is relevant in a “Remember Faces” block, but irrelevant in a “Remember Scenes” block). Then, we compared the fidelity of these representations following left IFG TMS to those following left post-central gyrus TMS (control site).

### Exploratory Searchlight MVPA Analyses

In an independent dataset in which participants did not undergo TMS (*n* = 24), a whole-brain Gaussian Naïve Bayes searchlight classifier (Pereira and Botvinick, 2011) was used to identify brain regions reliably representing each information code (e.g., *stimulus category* and *goal relevance*). Nine of these subjects later participated in the TMS experiment, but the data used in this exploratory searchlight analysis was separate from the data later analyzed for TMS effects.

**Figure 2:**
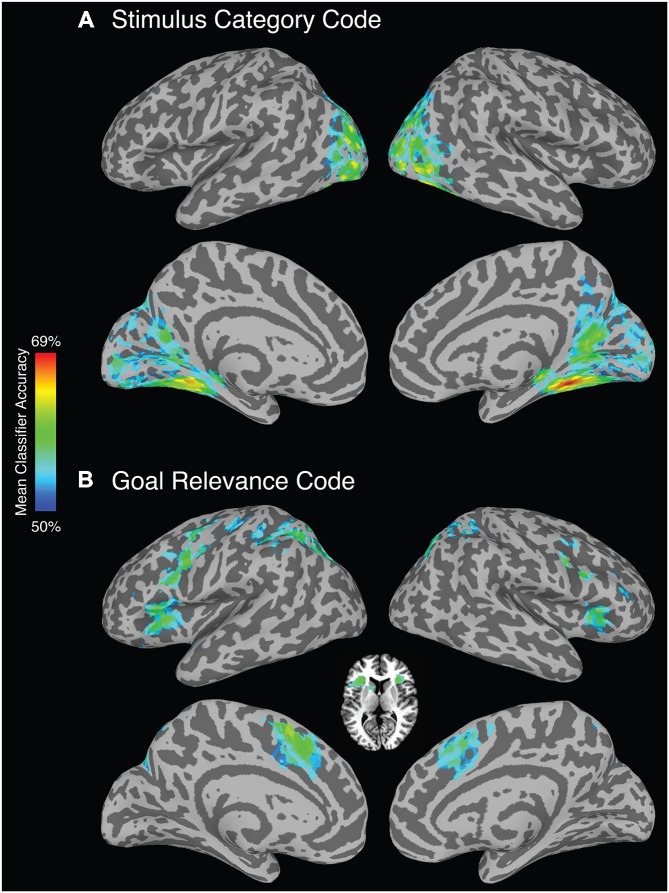
**Whole-brain searchlight analysis (FDR corrected, alpha *p* < 0.0001). (A)** Brain regions that reliably represent stimulus category. **(B)** Brain regions that reliably represent *goal relevance*. Axial slice depicts voxels identified in the bilateral anterior insula/frontal operculum and left caudate nucleus.

As predicted, a *stimulus category* code was reliably identified in extrastriate cortex, but also within primary visual cortex and parietal cortex. To identify category-selective ROIs to test for TMS effects, we selected voxels within these areas using a highly stringent FDR-corrected alpha level of 0.0001 (Figure 2A). Anatomical coordinates of these ROIs are presented in Table 2.

**Table 2:** **Anatomical locations and MNI coordinates of the centers of mass of clusters used as *stimulus category* functional ROIs**.

Anatomical region	MNI coordinates (mm)		
	*x*	*y*	*z*
Left fusiform gyrus	−30	−61	−4
Right fusiform gyrus	32	−58	−3

A *goal relevance* code was reliably identified in a bilateral set of regions including lateral and medial PFC, premotor cortex, superior parietal cortex, and striatum (Figure 2B). To identify goal-relevance ROIs to test for TMS effects, we selected voxels within these areas using a highly stringent FDR-corrected alpha level of 0.0001. Voxel clusters were identified in IFG, supplementary motor area, precentral sulcus/precentral gyrus, inferior parietal lobule, and angular gyrus, and left caudate nucleus. Anatomical coordinates of these ROIs are presented in Table 3.

**Table 3:** **Anatomical locations and MNI coordinates of the centers of mass of clusters used as *goal relevance* functional ROIs**.

	MNI coordinates (mm)		
Anatomical region	*x*	*y*	*z*
Bilateral supplementary motor area	−1	14	45
Left anterior insula/frontal operculum	−36	21	3
Left caudate nucleus	−15	9	7
Left precentral sulcus/precentral gyrus/inferior frontal junction	−43	0	38
Left inferior parietal lobule	−31	−55	45
Right anterior insula/frontal operculum	37	25	5
Right precentral sulcus/precentral gyrus/inferior frontal junction	48	9	34
Right angular gyrus	29	−59	44

### Effect of Left IFG TMS on *Stimulus Category* Code

#### ROI-Based Analyses

**Figure 3:**
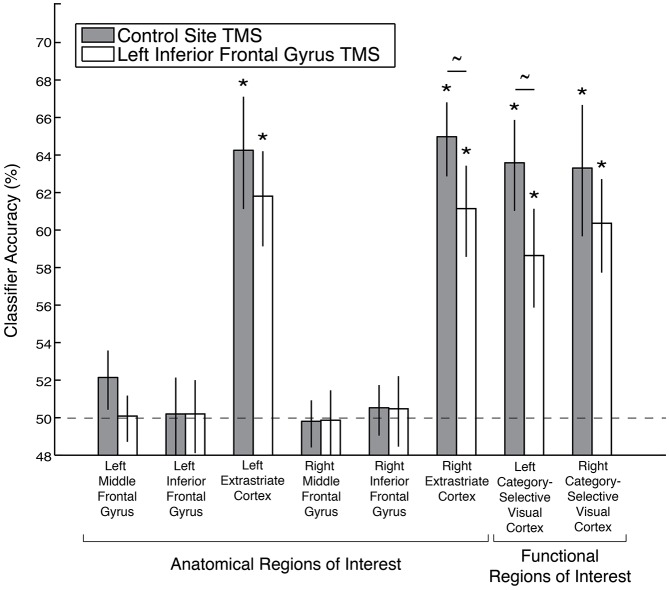
**Decoding accuracy of the *stimulus category* code following left IFG TMS, as compared to control TMS, in anatomical and functional ROIs.** Asterisks indicate significance after Bonferroni correction for multiple comparisons, and tildes indicate *p* < 0.05 without Bonferroni correction. Error bars depict ± standard error of the mean. Dashed line indicates chance classification accuracy.

A *stimulus category* code was reliably identified within functional ROIs defined from the whole-brain searchlight analysis following control site TMS (Figure 3). Mean classification accuracies were 63% in these ROIs in both hemispheres (both significant after Bonferroni correction for multiple comparisons; permutation test *p*’s < 0.02 corrected, *p*’s < 0.001 uncorrected). In addition, a *stimulus category* code was reliably identified in these ROIs after left IFG TMS [mean classification accuracies of 59% (left) and 60% (right), *p*’s < 0.02 corrected, *p*’s < 0.001 uncorrected]. While a small effect, decoding accuracy of the *stimulus category* code in the left visual cortex functional ROI was reduced by left IFG TMS (*p* = 0.01, uncorrected). Decoding accuracy of the *stimulus category* code in the right visual cortex functional ROI was not affected by left IFG TMS (*p* = 0.08, uncorrected), but the more restricted anatomical extrastriate ROI exhibited a significant decrease in *stimulus category* code decoding accuracy after IFG TMS (TMS effect: *p* = 0.03, uncorrected).

A *stimulus category* code was not reliably identified in the anatomical MFG or IFG ROIs after either control TMS (left MFG: *p* = 0.08; right MFG: *p* = 0.56; left IFG: *p* = 0.49; right IFG: *p* = 0.39, all *p*’s uncorrected) or after left IFG TMS (left MFG: *p* = 0.49; right MFG: *p* = 0.56; left IFG: *p* = 0.457; right IFG: *p* = 0.39, all *p*’s uncorrected, Figure 3). There were also no significant differences between the TMS conditions in these four anatomical ROIs (all *p*’s > 0.18, uncorrected).

#### Searchlight Analyses

**Figure 4:**
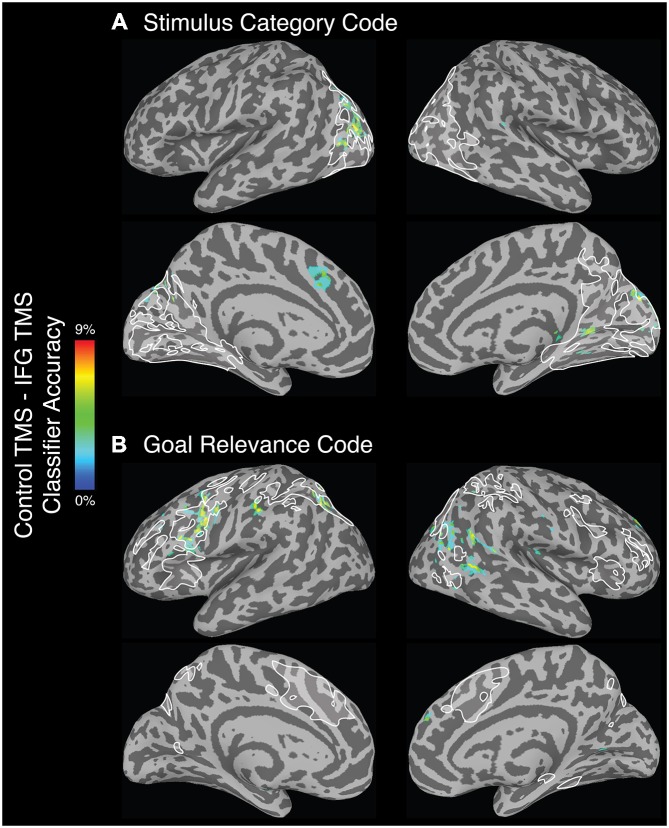
**Regions that exhibited a significant decrease in (A) *stimulus category* and (B) *goal relevance* code decoding accuracy following left IFG TMS, as compared to control TMS.** All depicted voxels are significant at the alpha (*p* < 0.005) level, and only voxel clusters larger than 99.5% of the null distribution of cluster sizes are shown here. White outlines depict the regions that showed above-chance classification in the exploratory searchlight analysis used to identify the *stimulus category* and *goal relevance* codes (see “Exploratory Searchlight MVPA Analyses” Section), voxelwise uncorrected *p* < 0.001.

Following left IFG TMS, the whole-brain searchlight analysis identified a number of significant clusters in bilateral occipital and parietal cortex, and left superior medial gyrus, that exhibited a significant decrease in *stimulus category* code decoding accuracy (Figure 4A). Anatomical coordinates of these regions are presented in Table 4. Voxels within left fusiform gyrus, intraparietal, middle occipital, and parieto-occipital sulci, and in the right calcarine sulcus and cuneus exhibited spatial overlap with the category-selective regions identified in the independent exploratory searchlight analysis for identifying the stimulus category code (Figure 2A). Voxels in the superior medial gyrus were not identified in the exploratory searchlight analysis.

**Table 4:** **Anatomical locations and MNI coordinates of the centers of mass of voxel clusters showing significant decreases in *stimulus category* code decoding accuracy after left IFG TMS**.

	MNI Coordinates (mm)		
Anatomical region	*x*	*y*	*z*
Left superior medial gyrus	−7	21	38
Left fusiform gyrus*	−40	−56	−9
Left intra-parietal sulcus*	−22	−68	27
Left middle occipital sulcus*	−39	−79	17
Left parieto-occipital sulcus*	−10	−80	37
Right calcarine sulcus*	24	−45	5
Right cuneus*	9	−84	36

*Indicate clusters within regions that also reliably represented the *stimulus category* code in a searchlight analysis on an independent, no-TMS dataset.

While it is unclear how to interpret *increases* in classification accuracy following IFG TMS as compared to control TMS, we found significant increases in *stimulus category* code decoding accuracy in the bilateral insula, right IFG, right superior temporal gyrus, and left middle temporal gyrus. In none of these regions was *stimulus category* reliably coded in the independent, no-TMS dataset used for functional ROI definition (see “Regions of interest—functional” Section ).

### Effect of Left IFG TMS on *Goal Relevance* Code

#### ROI-Based Analyses

**Figure 5:**
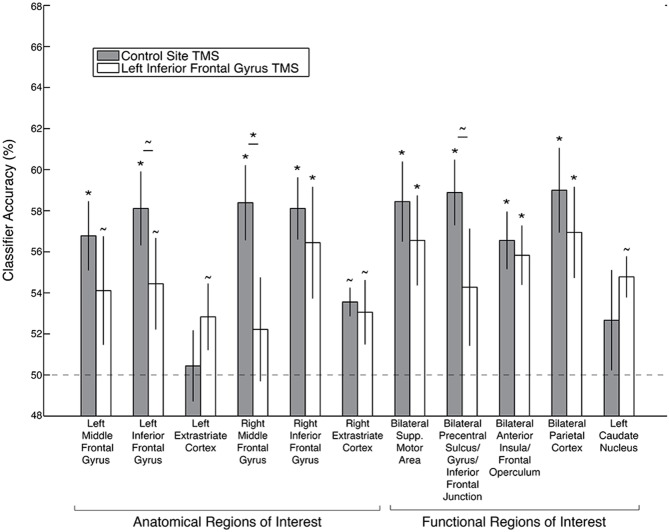
**Decoding accuracy of the *goal relevance* code following left IFG TMS, as compared to control TMS, in anatomical and searchlight ROIs.** Asterisks indicate significance after Bonferroni correction, and tildes indicate *p* < 0.05 without Bonferroni correction. Error bars depict ± standard error of the mean. Dashed line depicts chance classification accuracy.

A *goal relevance* code was reliably identified following control site TMS within all of the functional ROIs defined from the whole-brain searchlight analysis in an independent dataset: bilateral IFG, MFG, supplementary motor area, precentral sulcus/precentral gyrus/inferior frontal junction (IFJ), anterior insula/frontal operculum, parietal cortex, and left caudate nucleus (caudate *p* = 0.03, uncorrected, all other permutation test *p*’s < 0.02 corrected, *p*’s < 0.001 uncorrected; Figure 5). After left IFG TMS, however, the *goal relevance* decoding accuracy was significantly reduced, both in the left IFG (TMS effect: *p* = 0.04, uncorrected), right MFG (TMS effect: *p* < 0.01 corrected, *p* < 0.001 uncorrected) and the bilateral precentral sulcus/precentral gyrus/IFJ functional ROI (TMS effect: *p* = 0.01, uncorrected). We further examined the significant effect of left IFG TMS on the *goal relevance* code in the bilateral precentral sulcus/precentral gyrus/IFJ ROI by performing the classification analyses separately within each hemisphere. While *goal relevance* was represented with high reliability in the left and right ROIs both after control TMS and after left IFG TMS (all *p*’s < = 0.004 corrected), the left IFG TMS marginally reduced decoding accuracy in both hemispheres (left TMS effect: *p* = 0.07, right TMS effect: *p* = 0.05, both uncorrected).

Following left IFG TMS, there was no significant decrease in *goal relevance* decoding accuracy in the right IFG (TMS effect: *p* = 0.34), left MFG (TMS effect: *p* = 0.10), bilateral supplementary motor area (TMS effect: *p* = 0.19), bilateral anterior insula/frontal operculum (TMS effect: *p* = 0.31), left caudate nucleus (TMS effect: *p* = 0.82) or bilateral parietal cortex ROI (TMS effect: *p* = 0.17).

#### Searchlight Analyses

Following left IFG TMS, as compared to control site TMS, the whole-brain searchlight analysis identified several brain regions that exhibited a significant decrease in *goal relevance* decoding accuracy (Figure 4B). These regions were found throughout the frontal, parietal and occipital cortex (Table 5). Mirroring the ROI-based analysis, significant reductions were found in the left IFG, precentral sulcus, middle occipital gyrus/intra-parietal sulcus and right middle temporal gyrus, and calcarine gyrus. Anatomical coordinates of these regions are presented in Table 5.

**Table 5:** **Anatomical locations and MNI coordinates of the centers of mass of voxel clusters exhibiting significant decreases in *goal relevance* code decoding accuracy after left IFG TMS**.

	MNI Coordinates (mm)		
Anatomical region	*x*	*y*	*z*
Left inferior frontal gyrus*	−54	12	12
Left precentral sulcus/precentral gyrus*	−47	6	34
Left postcentral gyrus	−42	−21	33
Left middle occipital gyrus/intra-parietal sulcus*	−26	−62	40
Right superior frontal gyrus	16	47	29
Right middle temporal gyrus*	54	−58	18
Right calcarine gyrus*	33	−65	12

*Indicate clusters within regions that reliably represented the *goal relevance* code in a searchlight analysis of an independent, no-TMS dataset.

We found significant *increases* in *goal relevance* decoding accuracy in the right insula, left middle temporal gyrus, and left lingual gyrus, although none of these regions exhibited significant coding of *goal relevance* in the independent no-TMS dataset (see “Regions of interest—functional” Section ).

### Behavioral Analyses

Across both the “Remember Faces” and “Remember Scenes” conditions, participants performed the face/scene matching task with 92.9% mean accuracy after control site TMS. After left IFG TMS, mean accuracy was reduced to 90.1%. We tested for a brain-behavior relationship within the ROIs that showed a significant effect of TMS on decoding accuracy of the *stimulus category* code (left category-selective visual cortex functional ROI) and the *goal relevance* code (right MFG, left IFG, and bilateral precentral sulcus/IFJ), using an independent samples *t*-test on a median split of TMS-induced behavioral accuracy decrement (i.e., accuracy after control TMS minus accuracy after IFG TMS). While under-powered given the small number of subjects, no significant differences between the most- and least-impaired participants were found in the TMS effect on the *stimulus category* code in the left visual cortex functional ROI (*t*_(5.45)_ = 0.62, *p* = 0.56), or on the *goal relevance* code in the right MFG (*t*_(6.95)_ = −0.95, *p* = 0.37), left IFG (*t*_(4.02)_ = −0.69, *p* = 0.53), or bilateral precentral sulcus (*t*_(6.4)_ = 1.22, *p* = 0.27).

In our previous analysis of this dataset (Lee and D’Esposito, 2012), we found that increased activity in the right (non-disrupted) IFG after TMS predicted resistance to the behavioral impairment caused by TMS. To further clarify this result, we tested for a relationship between behavioral accuracy after IFG TMS and decoding accuracy of the *goal relevance* code in this region. Across the large right IFG anatomical ROI, we found a significant positive correlation, such that those participants who showed high accuracy on the task exhibited reliable coding of *goal relevance* in the right IFG (Spearman’s *rho* = 0.65, *p* = 0.04). As expected given the reduction of the *goal relevance* code in the MFG after left IFG TMS, there was no such relationship in either the left or the right MFG (left MFG: *rho* = 0.35, *p* = 0.36; right MFG: *rho* = 0.57, *p* = 0.11).

## Discussion

A growing body of evidence suggests that the prioritized processing and storage of information in VWM relies on top-down modulation of visual areas by the PFC (Miller and D’Esposito, 2005; Bressler et al., 2008; Sreenivasan et al., 2014a; D’Esposito and Postle, 2015). Here, we add causal evidence that the lateral PFC provides top-down signals that modulate the category-selectivity of visual cortex during VWM. In addition, we provide evidence that integration of an overarching task goal with incoming visual information is at least partially subserved by the left IFG, from which this information is likely transmitted to other regions responsible for further VWM processing.

In this study, we conducted a set of multi-voxel pattern analyses to identify brain regions that code for *stimulus category* and *goal relevance* during a face/scene matching task. Second, we determined how the *fidelity* of these codes (as indexed by multi-voxel pattern analysis classifier decoding accuracy) is affected by disruption of the lateral PFC. As predicted, we found that *stimulus category* information was represented most reliably in extrastriate cortex, extending to early visual cortex and posterior parietal cortex. After left IFG disruption, there was a moderate reduction in the fidelity of the *stimulus category* code within these regions in both hemispheres. This finding is consistent with two previous studies that investigated the remote effects of disrupted lateral PFC function on visual cortical activity during VWM. The first (Miller et al., 2011), found that disruption of PFC function, both with TMS in healthy participants and in patients with lateral PFC lesions due to stroke, reduces the distinctiveness of extrastriate cortex responses to face and scene stimuli. The second, using a different type of analysis of the data used in the present study (Lee and D’Esposito, 2012), also found that PFC disruption with TMS in healthy individuals causes a reduction in visual category selectivity in extrastriate cortex. Importantly, the participants for whom the lateral PFC disruption reduced the tuning of extrastriate responses to faces and scenes the most showed the greatest impairments in behavioral accuracy. While numerous correlational studies, both in humans (e.g., Gazzaley et al., 2004, 2007; Nee and Jonides, 2009; Tamber-Rosenau et al., 2011; Kuo et al., 2014) and in non-human primates (e.g., Freedman et al., 2003), have provided indirect evidence for top-down modulation of visual cortex by the PFC during VWM, the use of transient PFC disruption with TMS contributes important causal evidence for this model of cognitive control.

While the *stimulus category* code presumably arises largely as a result of “bottom-up” visual processing, the coding of *goal relevance* depends on the integration of a high-level task goal with bottom-up stimulus category information. This bridge between task goal and incoming visual information, while crucial for successful VWM performance, has not been well-characterized. In the current analyses, we found that the goal-relevance of incoming visual information, as determined by the current task set, was coded reliably in a distributed network of regions thought to be important for cognitive control, selective attention, and working memory (Dosenbach et al., 2006; Harding et al., 2015; also see Lückmann et al., 2014; for a review of these regions in attentional orienting in working memory). These goal-relevance regions included the IFG, MFG, precentral sulcus/IFJ, supplementary motor area, left striatum, and parietal and extrastriate cortices.

Following left IFG TMS, the fidelity of the *goal relevance* code decreased within that region, as predicted. In addition, the left IFG TMS also disrupted the *goal relevance* code in the MFG, suggesting that MFG relies on input from, or reciprocal communication with, the left IFG for the selective processing and maintenance of visual information. Previous functional connectivity analyses have suggested that the MFG plays a key role in VWM distractor resistance and in protecting items in memory from interference (Sakai et al., 2002; Postle, 2005), while the IFG may play a stronger role in determining the level of attention to allocate to incoming stimuli, based on task goals (Clapp et al., 2010). Considering these and the present findings, it is possible that the IFG is involved in determining whether an incoming stimulus is goal relevant, and gating information transfer to MFG accordingly, to aid in the protection of current items in memory from interference. Consistent with this proposed model (Feredoes et al., 2011) found that disruption of right MFG function with TMS during the presentation of distractors in a delayed recognition task caused increased activity in visual regions selective for the category of the remembered item.

After left IFG TMS, we also found a significant decrease in the fidelity of a *goal relevance* code within bilateral precentral sulcus/IFJ. A previous human fMRI/ERP study demonstrated that TMS to right IFJ before a similar delayed recognition task impaired task accuracy, and the size of the behavioral decrement was predicted by the degree to which top-down modulation of early visual cortex activity by the IFJ was impaired (Zanto et al., 2011). In addition, in a human MEG study, it was found that attention to different object categories induced gamma synchrony between the IFJ and the extrastriate regions most selective for those categories (Baldauf and Desimone, 2014). Moreover, the gamma activity in IFJ slightly preceded activity in extrastriate regions, which was interpreted as evidence that the IFJ directs visual processing via gamma synchrony with category-selective visual areas. Therefore, in the context of our results, it is possible that top-down modulation of visual areas by the lateral PFC is accomplished via processing of goal-relevance information in bilateral precentral sulcus/IFJ, from which goal-directed attention (Asplund et al., 2010) may be deployed to shape bilateral extrastriate cortical response selectivity (e.g., Chen et al., 2012). Further, it is likely that other brain regions, such as the frontal eye fields (e.g., Taylor et al., 2007), contribute additional top-down signals that aid VWM.

Finally, left IFG disruption did not significantly reduce the fidelity of the *goal relevance* code in the right IFG. However, participants who exhibited greater fidelity of the *goal relevance* code in this region after TMS performed the task with the highest accuracy. These findings are consistent with our original analyses of this dataset (Lee and D’Esposito, 2012). In that study, we found that increased functional connectivity between the right IFG and the right extrastriate cortex before TMS, and increased activity in the non-disrupted IFG after TMS, predicted resistance to the behavioral VWM impairment caused by TMS. Therefore, the current analysis provides additional insight into a potential compensatory mechanism, whereby reliable coding of *goal relevance* in a region homologous to the disrupted PFC area can provide protection against behavioral VWM impairment.

## Author Contributions

MD, AJ-WC, TGL, and ESL participated in study conception and design, TGL collected the data, ESL conducted the analyses, and ESL drafted the manuscript with critical revisions from TGL, AJ-WC, and MD.

## Funding

This work was supported by the National Institutes of Health Grants MH63901 and NS40813, the National Science Foundation Major Research Instrumentation Program BCS-0821855, and the VA Office of Rehabilitation Research and Development.

## Conflict of Interest Statement

The authors declare that the research was conducted in the absence of any commercial or financial relationships that could be construed as a potential conflict of interest.
